# STK11 loss leads to YAP1-mediated transcriptional activation in human KRAS-driven lung adenocarcinoma cell lines

**DOI:** 10.1038/s41417-023-00687-y

**Published:** 2023-11-15

**Authors:** Sean M. Lenahan, Hailey M. Sarausky, Paula Deming, David J. Seward

**Affiliations:** 1 Department of Pathology and Laboratory Medicine, University of Vermont College of Medicine, Burlington, VT USA; 2 Department of Biomedical and Health Sciences, University of Vermont College of Nursing and Health Sciences, Burlington, VT USA; 3 University of Vermont Cancer Center, Burlington, VT USA

**Keywords:** Non-small-cell lung cancer, Biomarkers

## Abstract

Serine Threonine Kinase 11 (STK11) loss of function (LoF) correlates with anti-PD-1 therapy resistance in patients with KRAS-driven lung adenocarcinoma (LUAD). The molecular mechanisms governing this observation remain unclear and represent a critical outstanding question in the field of lung oncology. As an initial approach to understand this phenomenon, we knocked-out (KO) STK11 in multiple KRAS-driven, STK11-competent human LUAD cell lines and performed whole transcriptome analyses to identify STK11-loss-dependent differential gene expression. Subsequent pathway enrichment studies highlighted activation of the HIPPO/YAP1 signaling axis, along with the induction of numerous tumor-intrinsic cytokines. To validate that YAP1-mediated transcriptional activation occurs in response to STK11 loss, we pursued YAP1 perturbation as a strategy to restore an STK11-competent gene expression profile in STK11-KO LUAD cell lines. Together, our data link STK11 loss with YAP1-mediated transcriptional activation, including the upregulation of immune-evasion promoting cytokines IL-6, CXCL8 and CXCL2. Further, our results raise the intriguing possibility that YAP1 antagonism may represent a therapeutic approach to counter anti-PD-1 therapy resistance in STK11-null, KRAS-driven LUADs by modulating tumor-intrinsic gene expression to promote a “hot” tumor immune microenvironment.

## Introduction

More people die in the US each year from lung cancer than breast, colorectal, and prostate cancers combined [1]. The overall prognosis for patients with lung cancer remains poor, complicated by the fact that >40% of patients are diagnosed at an advanced stage [1]. Immune check-point inhibitors, including anti-PD-1 monoclonal antibodies, are now utilized as first line therapy when treating lung adenocarcinoma (LUAD) patients, though our ability to predict which patients will benefit remains limited [2, 3]. Recent clinical studies have linked anti-PD-1 therapy resistance with tumor genotype. Specifically, KRAS-driven LUADs with concomitant loss of Serine/Threonine Kinase 11 (STK11) exhibit a dramatic decrease in therapeutic efficacy relative to tumors harboring intact STK11 [4]. The molecular mechanism(s) underlying this correlation remain unknown.

STK11 functions in a heterotrimeric complex with the pseudo-kinase STRADa and the scaffolding protein MO25 where it regulates numerous intracellular signaling networks impacting metabolism, proliferation, transcription and cell morphology [5]. In LUAD, somatic alterations in STK11 are surpassed in frequency by only one other tumor suppressor, TP53 [6, 7]. A growing body of work reveals STK11 regulates myriad biologic processes and suggests the scope of STK11-dependent regulation is vastly underappreciated [8–11]. Previous studies using inducible mouse models of Kras-driven lung cancer have reported Stk11 deficiency correlates with altered tumor-intrinsic cytokine expression [12]. We therefore reasoned, as others have [4, 12], that increased production and secretion of tumor-intrinsic cytokines might alter recruitment of immune cells to the tumor microenvironment, thereby promoting immune evasion and contributing to anti-PD-1 therapy resistance. However, instead of focusing on the tumor-extrinsic processes, we chose to investigate which signaling pathways downstream of STK11 loss drive these tumor-intrinsic transcriptional changes.

Using multiple KRAS-driven human LUAD cell lines, our work demonstrates that STK11 loss results in altered tumor-intrinsic cytokine expression, including but not limited to IL-6, CXCL8 and CXCL2. In addition, we identify significant enrichment of a Yes Associated Protein 1 (YAP1) transcriptional signature [13] upon STK11 disruption, which encompasses a variety of cytokines, chemokines, and extracellular matrix proteins [14]. YAP1 is the major downstream effector of the HIPPO signaling cascade, a pathway that regulates organ size during development [15]. YAP1-mediated transcriptional activation is controlled in part via its cytosolic sequestration; a kinase-dependent process controlled by activation of the HIPPO pathway [16]. Whether HIPPO signaling is impacted by STK11 remains incompletely addressed, but recent publications suggest a connection exists [13, 17, 18]. Our data support this link via restoration of STK11-loss-dependent transcriptional profiles upon either genetic ablation or pharmacologic inhibition of YAP1. The work we present situates the HIPPO/YAP1 axis as a signaling cascade downstream of STK11 that modulates tumor-intrinsic cytokine expression. Further, we speculate YAP1 antagonism may represent a synergistic strategy to sensitize patients with KRAS-driven LUADs lacking STK11 to anti-PD-1 therapy by promoting a “hot” tumor immune microenvironment.

## Results

### STK11 loss alters tumor-intrinsic cytokine expression

**Fig. 1:**
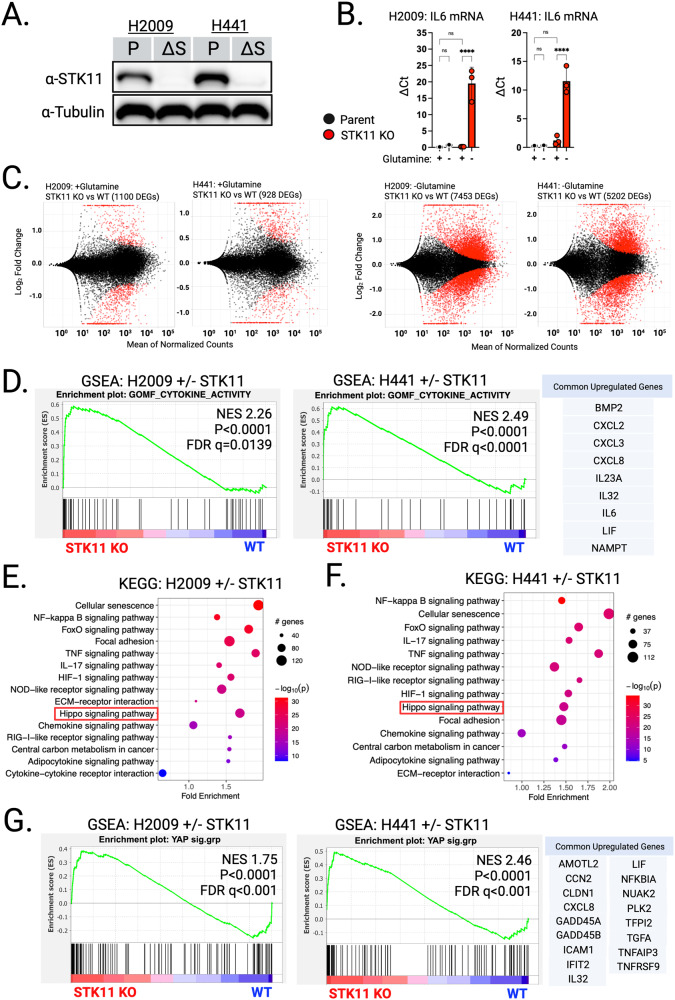
STK11 loss alters the transcriptional response to nutrient stress in human KRAS-driven lung adenocarcinoma cell lines. **A** Western blot analysis confirming knock-out of STK11 (ΔS) in NCI-H2009 and NCI-H441 parent (P) cell lines. **B** IL-6 mRNA expression in parent versus STK11-KO cell lines grown in standard media (+Glutamine) or glutamine depleted media (-Glutamine). Gene expression normalized to PSMB4. Data presented as mean ± SD (*N* = 3). **C** MA plots generated from RNA-seq analysis demonstrate few differentially expressed genes (DEGs) between parent (WT) and STK11-KO cell lines when grown in standard media (+Glutamine; 1100 DEGs for H2009, 928 DEGs for H441). In contrast, the same cells grown in glutamine depleted media exhibit massive increases in DEGs in both cell lines (−Glutamine; 7453 DEGs for H2009, 5202 DEGs for H441). **D** GSEA performed on DEGs from each cell line pair grown in the absence of glutamine identified “Cytokine Activity” (GO: 0005125) as significantly enriched and positively correlated with STK11 loss. Upregulated genes from the “Cytokine Activity” list shared across H2009 and H441 STK11 KO cell lines are listed. **E**, **F** KEGG Pathway Enrichment Analysis performed on DEGs from H2009 and H441 cell lines comparing parent and STK11-KO cells following glutamine depletion. As expected, pathways related to cytokine signaling were identified. Notably, the “Hippo signaling pathway” (red box) was significantly enriched in both cell lines. **G** GSEA performed on DEGs using a curated YAP1 transcriptional signature demonstrates a strong positive correlation with STK11 loss in both cell lines suggesting YAP1 transcriptional activation occurs when cells experience glutamine depletion in the absence of STK11. Upregulated genes from the curated YAP1 signature shared across H2009 and H441 STK11 KO cell lines upon glutamine depletion are listed. ****p < 0.0001 was calculated by two-way ANOVA and the Tukey test in (**B**).

We knocked-out STK11 in three genetically independent human KRAS-driven LUAD cell lines that normally harbor intact STK11 alleles: NCI-H2009, NCI-H441 and NCI-H1792. STK11 loss was validated by Western Blot analysis (Fig. 1A and Supplemental Data). Based on studies reporting a correlation between Stk11 loss and Il-6 upregulation in mouse models of Kras-driven lung cancers in vivo [12], we compared IL-6 expression between STK11 WT (aka “Parent”) and matched STK11-KO human LUAD cells using qRT-PCR. Unexpectedly, under standard culture conditions no difference in IL-6 expression was detected between the Parent and STK11-KO cells (Fig. 1B, “+Glutamine”). However, significant STK11-loss-dependent IL-6 upregulation was observed when cells were cultured under conditions of nutrient stress, achieved via glutamine depletion (Fig. 1B, “−Glutamine”). The rationale for evaluating nutrient stress as a variable was based on evidence that STK11 functions as a nutrient sensor to regulate metabolic homeostasis [19–21]. We reasoned STK11 loss might be irrelevant when cells are grown in standard media as nutrients are in excess. Given that tumor microenvironments in vivo are characterized by nutrient stress [22–24], we used glutamine depletion to simulate nutrient-deprivation in vitro.

Next, to comprehensively characterize STK11-loss-dependent transcriptional changes, we expanded our analyses and performed whole transcriptome sequencing comparing standard media to glutamine depletion. In standard media, relatively few genes differed between parent and STK11-KO cells (Fig. 1C, +Glutamine; H2009: 1100 DEGs, H441: 928 DEGs). In contrast, when comparing both H2009 and H441 parent lines with their paired STK11-KO lines following glutamine depletion we identified 7453 and 5202 differentially expressed genes (DEGs) respectively (Fig. 1C; −Glutamine). This marked STK11-loss-dependent transcriptional impact indicates STK11 plays a critical and generalizable role in regulating transcription in response to nutrient stress. We then performed Gene Set Enrichment Analysis (GSEA) [25] on the DEGs for both H2009 and H441 cell lines and found significant associations between STK11 loss and altered tumor-intrinsic cytokine signaling, specifically upregulation of genes within the Gene Ontology (GO) term “Cytokine Activity” (GO: 0005125) (Fig. 1D). Of the upregulated genes in this curated list, 9 were shared between the H2009 and H441 cell lines, suggesting overlapping regulatory pathways. Intriguingly, these overlapping genes consist of effectors previously associated with cancer progression, immune evasion, and therapy resistance [26–28]. For example, both IL-6 and CXCL8 are reported to be elevated in KRAS-driven STK11-null LUADs and proposed to promote tumor immune evasion [29–31]. Similarly, CXCL2 is known to drive neutrophil recruitment, a phenotype associated with “cold” tumor immune microenvironments [27]. Finally, BMP2 expression is correlated with metastatic burden and STK11 loss in lung cancer and mediates activation of SMAD transcription factors [32, 33], which are known YAP1 binding partners [34].

### YAP1 transcriptional activation occurs in LUAD cell lines lacking STK11

In addition to GSEA, we also performed pathway enrichment using the Kyoto Encyclopedia of Genes and Genomes (KEGG) database [35]. This approach revealed several significantly enriched networks in STK11-KO cells relative to matched parental lines (Fig. 1E, F). Consistent with prior published reports, both focal adhesion and HIF-1 pathways were over-represented in cells lacking STK11 [36, 37]. In addition, NF-kappa B signaling, TNF signaling, chemokine signaling and HIPPO signaling were significantly enriched in STK11-KO cells. We chose to focus on the HIPPO pathway as STK11 has previously been implicated in HIPPO regulation via direct activation of MARK family kinases and subsequent modulation of YAP1 activity [13]. YAP1-mediated transcriptional activation is controlled in part via cytosolic sequestration; a kinase-dependent process regulated by activation of the HIPPO cascade [16]. Utilizing a curated list of YAP1 transcriptional target genes [13] we repeated GSEA and found a significant positive correlation between STK11 loss and enhanced expression of YAP1 target genes in both the H2009 and H441 cell lines (Fig. 1G).

### STK11 loss correlates with increased YAP1 protein levels

**Fig. 2:**
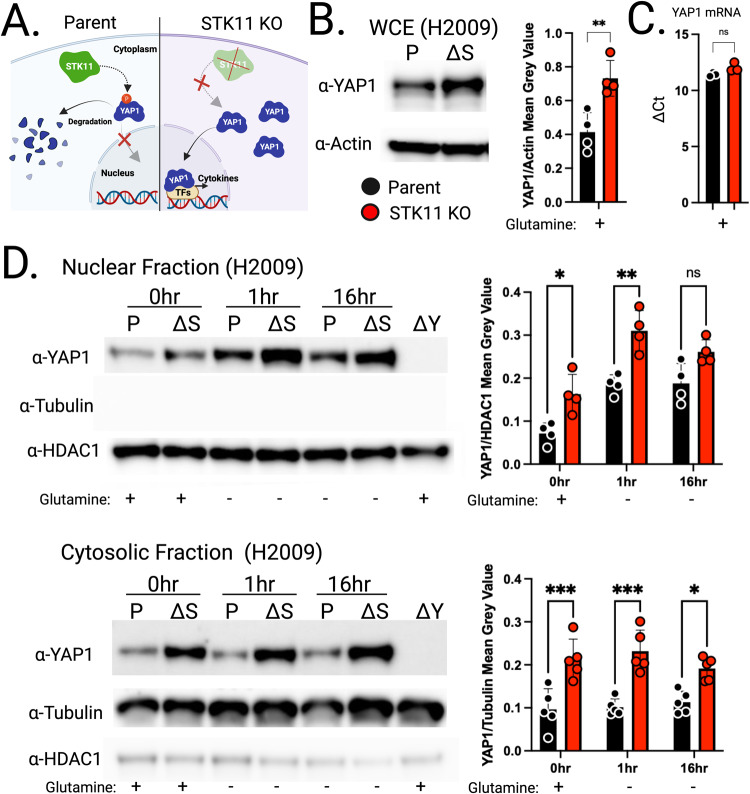
STK11 loss leads to increased YAP1 protein abundance in human KRAS-driven lung adenocarcinoma cell lines. **A** We posit STK11, either directly or indirectly, contributes to YAP1 cytoplasmic sequestration and degradation. If true, STK11 loss should lead to enhanced YAP1 protein accumulation and potentially increased transcriptional activity. **B** Western blot analysis targeting YAP1 in whole cell extracts (WCE) from H2009 parent (P) versus H2009 STK11 KO (ΔS) cells results in a ~2-fold increase in YAP1 protein. Data presented as mean ± SD (*N* = 4). **C** YAP1 qRT-PCR analysis argues the difference in YAP1 protein abundance is not due to enhanced YAP1 gene expression. Data presented as mean ± SD (*N* = 3). **D** Western blot analysis performed on nuclear and cytoplasmic fractions isolated from H2009 parent (P) or STK11 KO (ΔS) cells support the whole cell extract data showing enhanced YAP1 protein abundance in the absence of STK11. Nuclear fraction data presented as mean ± SD (*N* = 4). Cytoplasmic fraction data presented as mean ± SD (*N* = 5). *p < 0.0332, **p < 0.0021, ***p < 0.0002 was calculated by Student’s *t* Test (**B**, **C**) or two-way ANOVA and Tukey test in (**D**).

STK11 has previously been proposed to indirectly modulate the HIPPO/YAP1 axis via MARK activation, ultimately promoting YAP1 sequestration and degradation [13]. We therefore hypothesized that STK11 loss would result in increased YAP1 protein due to enhanced protein stabilization (Fig. 2A). Western blot analysis comparing whole cell extracts from parent and STK11-KO LUAD cell lines support this assertion, showing a ~2-fold increase in relative YAP1 abundance (Fig. 2B), an observation supported by prior studies in mice [13]. Interestingly, this difference occurs only at the protein level, as YAP1 transcript levels remain unchanged, supporting our hypothesis that STK11 loss results in YAP1 protein stabilization (Fig. 2C). Nuclear and cytosolic fractionation analyses further demonstrate that increased YAP1 protein levels are not isolated to either compartment but increased throughout cells lacking STK11. Upon glutamine deprivation, we observed increased YAP1 nuclear translocation in both parent and STK11-null cells, though the increase was more pronounced in the STK11-null cells (Fig. 2D). This data supports an STK11-dependent impact on global YAP1 protein abundance, including nuclear localization, which we posit drives changes in YAP1-mediated gene expression (Figs. 1G and 2A).

### YAP1 antagonism partially restores cytokine expression profiles in STK11 deficient cells

**Fig. 3:**
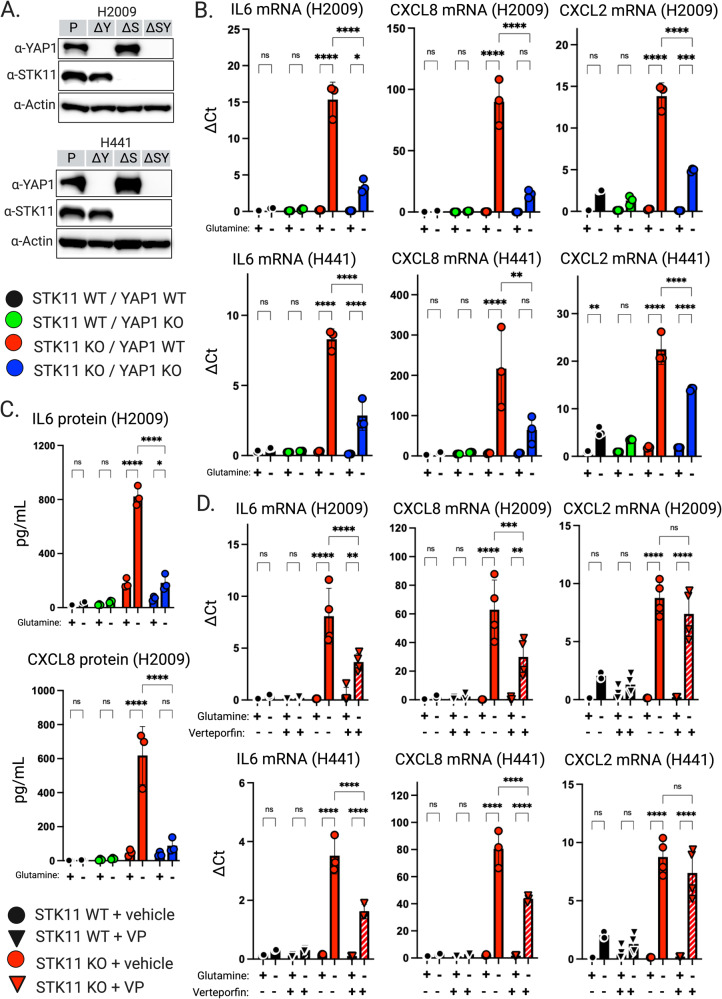
YAP1 perturbation blunts STK11-loss-dependent cytokine induction upon glutamine depletion in human KRAS-driven lung adenocarcinoma cell lines. **A** Western blot analysis confirming knockout of YAP1 (ΔY) in NCI-H2009 and NCI-H441 parent (P) and STK11-KO (ΔS) cell lines. The STK11/YAP1 double knockout lines are abbreviated as ΔSY. **B** IL-6, CXCL8, and CXCL2 qRT-PCR analysis demonstrates that upon glutamine depletion, the STK11-loss-dependent induction is blunted by the absence of YAP1. Expression normalized to PSMB4, and data presented as mean ± SD (*N* = 3). **C** IL6 and CXCL8 ELISAs performed on conditioned media from H2009 cell lines. Data presented as mean ± SD (*N* = 3). **D** qRT-PCR analysis of IL-6, CXCL8, and CXCL2 on cells treated with 1.5 mM verteporfin (VP) vs vehicle. Expression normalized to PSMB4, and data presented as mean ± SD (*N* = 3). *p < 0.0332, **p < 0.0021, ***p < 0.0002, ****p < 0.0001 was calculated by two-way ANOVA and Tukey test in (**B**, **C**) or three-way ANOVA and Tukey test in (**D**).

To validate our pathway analyses we reasoned we could inhibit STK11-loss-dependent cytokine induction following glutamine depletion by blocking the downstream signaling networks responsible. To examine the role of YAP1 in driving this phenotype, we engineered STK11/YAP1 double knockouts in both H2009 and H441 LUAD cell lines (Fig. 3A). Our data demonstrate significantly less IL-6, CXCL8 and CXCL2 expression in the STK11/YAP1 double KO lines compared with STK11-KO lines following glutamine depletion (Fig. 3B). Importantly, these changes were mirrored by levels of secreted IL-6 and CXCL8 protein levels measured by ELISA (Fig. 3C). YAP1-KO alone had no impact on expression of these cytokines, regardless of glutamine availability, demonstrating the necessity of STK11 loss in producing this phenotype (Fig. 3B).

After establishing YAP1 functions downstream of STK11 and is at least in part responsible for the increased cytokine expression occurring in STK11-KO cells following glutamine depletion, we next sought to phenocopy YAP1 KO via pharmacologic antagonism of YAP1 with verteporfin (VP) [38]. One mechanism by which VP is known to alter YAP1 activity occurs via physically disrupting the interaction between YAP1 and members of the TEAD transcription factor family [38]. Our data clearly show that the STK11-loss-dependent upregulation of IL-6 and CXCL8 upon glutamine depletion is blunted by VP treatment (Fig. 3D). Interestingly, this affect does not extend to CXCL2 (Fig. 3D). Together these results support CXCL8 and IL-6 expression are likely regulated, at least in part, by YAP1/TEAD interactions. The fact that CXCL2 expression is reduced upon YAP1 genetic ablation, but not VP treatment, was unexpected and suggests YAP1’s impact on CXCL2 expression may be independent of TEAD. YAP1 is known to interact with many transcription factors, including SMAD family members and the b-catenin/TBX5 complex [34]. We think it likely that YAP1’s impact on CXCL2 expression relies on a transcription factor other than a TEAD family member, which is why genetic ablation of YAP1 results in altered expression, whereas TEAD dissociation with VP does not. Whether this definitively explains the discrepancy in our CXCL2 data awaits further investigation but remains a favored hypothesis.

### YAP1 deletion restores gene expression profiles in STK11 deficient cells

**Fig. 4:**
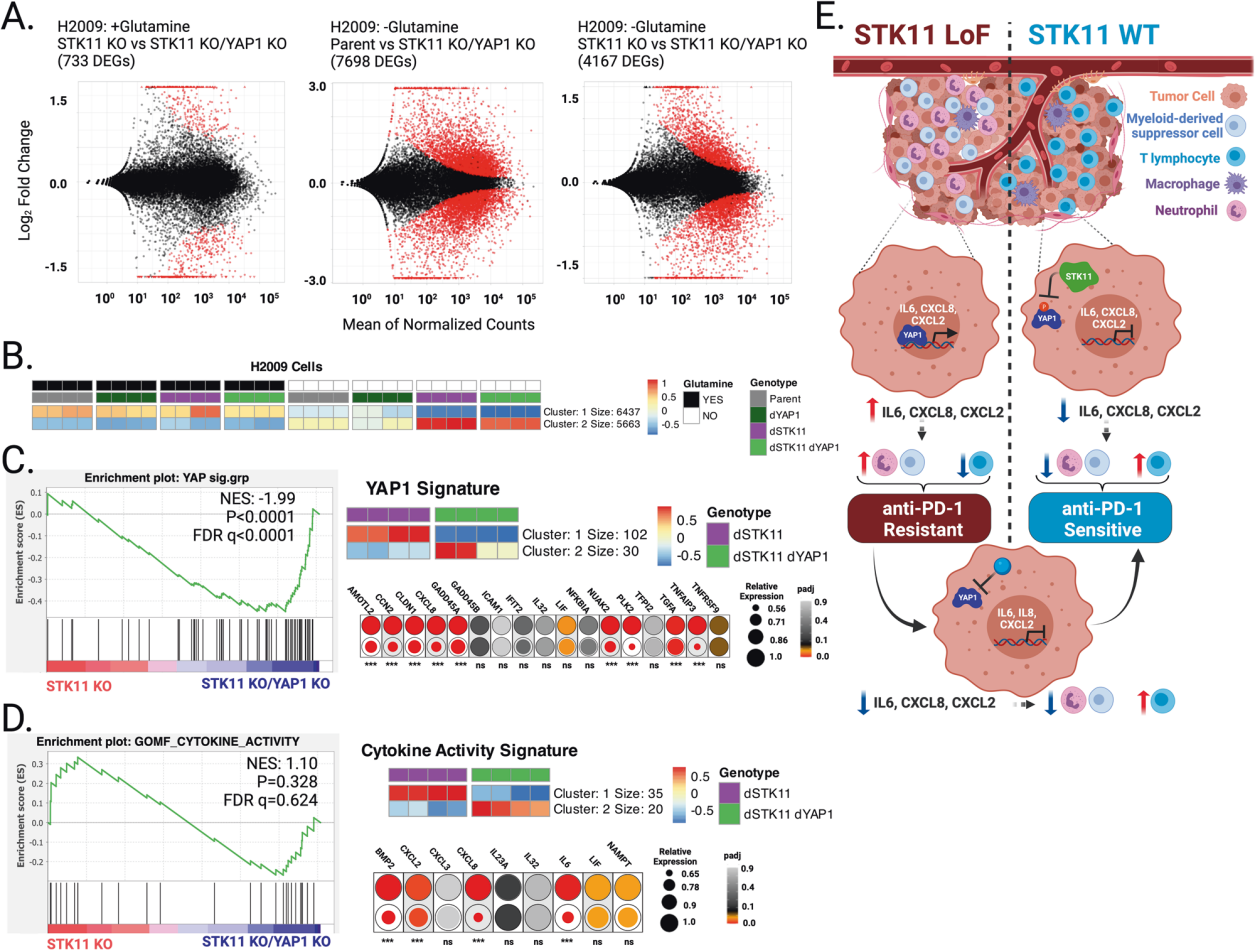
Global transcriptional analysis in STK11/YAP1 double KO cells indicate blunting of the STK11-loss-dependent expression signatures following glutamine depletion in STK11-null KRAS-driven lung adenocarcinoma cell lines. **A** MA-Plots generated from RNA-seq data contrast the number of differentially expressed genes in H2009 cell lines upon glutamine depletion. As expected, few DEGs are identified between STK11 KO and STK11/YAP1 double KO cells when cultured with glutamine (+Glutamine; 733 DEGs). A similar number of DEGs were detected in the STK11/YAP1 double KO compared with the parent line when grown in glutamine depleted media (−Glutamine, 7698 DEGs) as were seen in the STK11 KO (Fig. 1C, −Glutamine; 7453 DEGs). When the STK11/YAP1 double KO cells are compared directly with STK11 KO cells in the absence of glutamine, 4167 DEGs are detected. **B** *K*-means clustering of all mapped transcripts highlights genes that are induced upon glutamine depletion in STK11 KO cells, but whose induction is blunted in STK11/YAP1 double KO cells (Cluster 2, Red vs Orange). This group represents candidate YAP1-transcriptional targets. **C** GSEA performed on DEGs identified between STK11 KO and STK11/YAP1 double KO cells using the curated YAP1 signature gene list results in a strong negative correlation indicating reduced expression in the absence of YAP1. K-means clustering of the YAP1 gene signature supports this assertion (Cluster 1, Red vs Blue). Dot plot visualization of the 17 genes shared between H2009 and H441 cells (Fig. 1G) indicates the magnitude of expression blunting that occurs in the absence of YAP1. **D** GSEA performed on DEGs identified between STK11 KO and STK11/YAP1 double KO cells using the gene ontology cytokine activity list demonstrates no significant correlation, in line with a blunted response due to YAP1 loss. *K*-means clustering of the cytokine activity signature supports this assertion (Cluster 1, Red vs Blue). Dot plot visualization of the 9 genes shared between H2009 and H441 cells (Fig. 1D) indicates the magnitude of expression blunting that occurs in the absence of YAP1. **E** Proposed model linking the tumor-intrinsic role of an STK11/YAP1 axis with altered transcriptional profiles in KRAS-driven, STK11-null LUADs that promote a “cold” tumor immune microenvironment, potentiating anti-PD-1 therapy resistance. Our data support targeting YAP1 as a strategy to foster a “hot” tumor immune microenvironment, thereby sensitizing patients to anti-PD-1 therapy. ***p < 0.0001 reflects the *p*adj values attained by the Wald test and corrected for multiple testing using the Benjamini and Hochberg method within DESeq2.

To define the transcriptome-wide impact of YAP1 KO in STK11 deficient cells, we performed RNA-seq on H2009 cells following 24 h in either standard or glutamine depleted media. In standard media, few genes differed between STK11-KO and STK11/YAP1 double KO cells (Fig. 4A, +Glutamine; 733 DEGs). Compared with the H2009 parent line, similar numbers of DEGs were detected in the STK11/YAP1 double KO as were seen in the STK11 KO when grown in the absence of glutamine (Fig. 4A, −Glutamine; 7698 DEGs vs Fig. 1C, −Glutamine; 7453 DEGs).

However, when the STK11/YAP1 double KO cells are compared directly with STK11 KO cells in the absence of glutamine, 4167 DEGs were detected (Fig. 4A, −Glutamine; 4167 DEGs). If YAP1 loss had no impact, we would predict no DEGs identified between these two conditions. The DEGs detected represent genes that still change upon glutamine depletion, but the magnitude of that change is significantly reduced in the absence of YAP1 indicating these genes are candidates for YAP1-mediated regulation. *K*-means clustering of genes differentially expressed between STK11-KO and STK11/YAP1 double KO cells revealed a large group of genes that, while still induced by glutamine depletion, were repressed relative to the induction observed in STK11-null/YAP1-competent cells (Fig. 4B; cluster 2, Red v Orange). GSEA performed on DEGs identified between H2009 STK11-KO and STK11/YAP1 double KO cells using the previously described curated YAP1 gene signature demonstrated a significant negative correlation, indicating gene repression in STK11/YAP1 double KO cells relative to STK11-KO/YAP1-intact cells (Fig. 4C). Specifically, 102 genes within the curated YAP1 signature exhibited reduced expression upon YAP1 ablation in STK11-KO cells, highlighted by dot plot analysis of the 17 genes identified in Fig. 1G, which show overlap in gene induction between H2009 and H441 cells upon STK11 ablation (Fig. 4C). We posit those genes demonstrating significant reduction in expression are regulated in part by YAP1. We also performed GSEA using the cytokine activity signature (GO: 0005125) previously described (Fig. 1D) and observed repression of 35 member genes upon YAP1 ablation in STK11-KO cells (Fig. 4D). Again, dot plot analysis highlights repression of a subset of these genes following YAP1 deletion in H2009 cells lacking STK11 (Fig. 4D). Taken together, these data support YAP1 antagonism as a strategy to curb expression of key genes, including immunomodulatory cytokines, in KRAS-driven STK11-null LUADs. We speculate a similar response in vivo would aid in transitioning immunologically “cold” tumor immune microenvironments to “hot”, potentiating the effectiveness of checkpoint inhibitor therapies such as anti-PD-1 monoclonal antibodies (Fig. 4E).

## Discussion

LUAD patients with KRAS-driven/STK11-null tumors have poor response rates to anti-PD-1 therapy and decreased overall survival compared to patients with LUAD driven by KRAS mutations alone [4]. Somatic STK11 disruption is common in KRAS-driven LUADs (~10–15%), but rare in other KRAS-driven adenocarcinomas (pancreatic ~1.5%, colorectal ~1.2%). As a result, it is currently unclear whether STK11-dependent anti-PD-1 therapy resistance generalizes across KRAS-driven tumors or represents a LUAD specific phenomenon. Addressing this important question will require additional study.

Our data identifies the transcriptional effector YAP1 as impacting tumor-intrinsic gene expression in STK11-deficient, KRAS-driven human LUAD cell lines. This response is particularly robust upon glutamine depletion, a condition commonly found in lung tumor microenvironments in vivo [22–24]. Importantly, many immunomodulatory cytokines are among those genes prominently affected by STK11 loss, including IL-6, CXCL8 and CXCL2 [27, 29–31]. We demonstrate that YAP1 perturbation, both genetic and pharmacologic, blunts the STK11-loss-dependent induction of these immunomodulatory cytokines. Together our findings support targeting YAP1 as a strategy to block progression of the anti-PD-1 therapy resistance phenotype reported in KRAS-driven/STK11-null LUADs [4].

Understanding the mechanistic links between tumor genotype and therapy response has been essential to developing targeted cancer therapies, but this paradigm has primarily been limited to direct small molecule-mediated disruption of tumor-centric oncogenic protein function. Our work highlights links between tumor genotype-specific transcriptional signatures and putative paracrine signaling within the tumor microenvironment. Importantly, the transcriptome parallels we document are independent of the specific oncogenic KRAS variant present, as each cell line harbors a unique missense change at the G12 position (NCI-H441: KRAS p.G12V; NCI-H2009: KRAS p.G12A; NCI-H1792: KRAS p.G12C). Further, oncogenic variants at the G12 position comprise ~90% of the KRAS variants documented in human KRAS-driven LUADs, supporting the clinical relevance of our analyses. We speculate these genotype-dependent, tumor-intrinsic changes impact anti-PD-1 therapy resistance and immune-evasion by disrupting immune effector recruitment to the tumor immune microenvironment. Critically, we posit these phenotypes provide a therapeutic opportunity to synergize with current treatment regimes. While VP treatment did successfully blunt expression of IL-6 and CXCL8 in STK11-KO cells, it is far from an ideal YAP1 inhibitor due to its low solubility, stability, and off-target effects [39]. Intriguingly, the HIPPO/YAP1 axis, long considered undruggable, has recently been targeted via inhibition of TEAD in a phase 1 clinical trial (NCT04665206), and emerging projects are focused on manipulating YAP1 activity by regulating its post-translational modifications as an avenue to block its increased transcriptional activity [40].

Finally, YAP1 is not the only effector protein whose activity is altered downstream of STK11 loss in KRAS-driven LUADs. While we document many YAP1 target genes as induced upon glutamine depletion in STK11-KO cells, the expression of those genes is not completely restored to STK11 WT levels upon YAP1 loss. Further, there are many genes that show no expression change following YAP1 disruption (e.g., IL-32, CXCL3, IL23A; Fig. 4D), indicating the existence of YAP1-independent pathways downstream of STK11. Identifying these additional signaling cascades is the focus of ongoing work in our laboratory and we anticipate their discovery will offer novel strategies to counter anti-PD-1 therapy resistance related to STK11 loss in KRAS-driven LUAD.

### Supplementary information

Supplemental Data

## Data Availability

The data that support the findings of this study are available from the corresponding author upon reasonable request.
