# Supplementary material for: STK11 loss leads to YAP1-mediated transcriptional activation in human KRAS-driven lung adenocarcinoma cell lines
Source: Cancer Gene Ther. 2023 Nov 15;31(1):1–8. doi: 10.1038/s41417-023-00687-y (PMC10794139; doi:10.1038/s41417-023-00687-y)
Supplement: Supplementary file 1 — Supplemental Data [file 41417_2023_687_MOESM1_ESM.docx]

**Lenahan et al, Supplemental Information**

**Supplemental Data**

1. Anti-STK11 western blot showing STK11 KO from NCI-H1792 cells (attached supplemental figure 1).

Supplemental Figure 1:


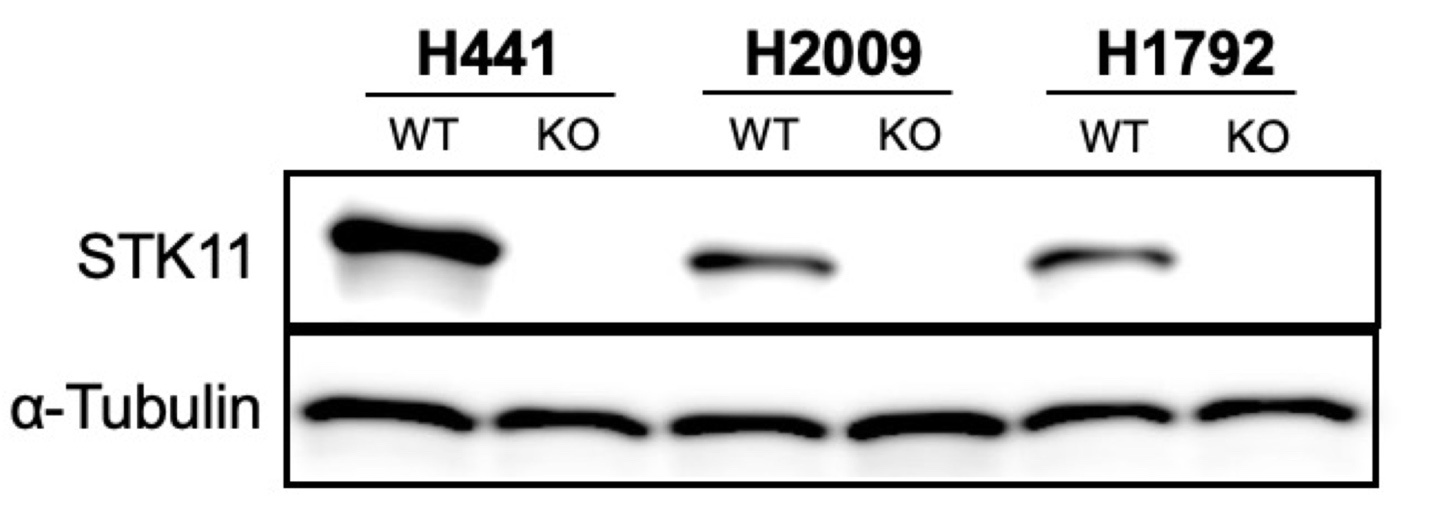


Supplemental Figure 1: STK11 Western blot demonstrating successful KO in multiple cells lines.

**Material and Methods**

**Cell culture and treatment**

NCI-H2009 (ATCC# CRL-5911; KRAS p.G12A), NCI-H441 (ATCC # HTB-174; KRAS p.G12V), and NCI-H1792 (ATCC # CRL-5895; KRAS p.G12C) were purchased new directly from ATCC, authenticated by STR, and tested monthly for mycoplasma by PCR. Cells were cultured in RPMI 1640 (Corning, 10-041-CV) containing 10% fetal bovine serum (Corning, 35-011-CV) at 37 C and 5% CO2. The following reagents were used for cell treatments: RPMI 1640 without L-Glutamine (Corning, 15-041-CV), Verteporfin (Sigma-Aldrich, SML0534), and DMSO (Fisher, BP232-1). Verteporfin was dissolved in DMSO at a concentration of 2.78 mM and stored at –20 C. Additional data for each of the cell lines is openly accessible via ATCC (atcc.org) or the Broad cell line encyclopedia (depmap.org). All cell based experiments were designed to meet or exceed the practical minimum sample number of 3 biologic replicates per condition.

**CRISPR-Cas9 gene Knockout**

Cells were seeded at 0.4-0.6 x 10^6 cells/well in a 6 well plate (Corning). Next day, cells were transfected with the pSPCas9(BB)-2A-GFP (Addgene, PX458) vector expressing Cas9, the sgRNA, and GFP using Fugene HD (Promega, E2311) following the manufacturer’s protocol. sgRNA’s targeting STK11 and YAP1 were designed and purchased using Integrated DNA Technologies (IDT) online tools. Single transfected cells were sorted into 96 well plates based on GFP expression using the BD FACS AriaIII in the University of Vermont Larner College of Medicine Flow Cytometry and Cell Sorting Facility (RRID:SCR_022147). Once expanded, clones were screened for successful knockout of STK11 or YAP1 by targeted locus-specific PCR and Sanger sequencing. Knockout was further validated by western blot analysis.

STK11 KO guide RNA v1 - CACCGCCACCGCATCGACTCCACCG

STK11 KO guide RNA v1 Complement - AAACCGGTGGAGTCGATGCGGTGGC

STK11 KO guide RNA v2 - CACCGGTTGCGAAGGATCCCCAACG

STK11 KO guide RNA v2 Complement - AAACCGTTGGGGATCCTTCGCAACC

hSTK11 GCD v1 FWD primer - GGGACTGACGTGTAGAACAATC

hSTK11 GCD v1 REV primer - GGAAGGAAGACAGAACCATCA

YAP1 KO guide RNA v1 - CACCGTGCACGATCTGATGCCCGG

YAP1 KO guide RNA v1 Complement - AAACCCGGGCATCAGATCGTGCAC

YAP1 KO guide RNA v2 - CACCGGTCGGTCTCCGAGTCCCCG

YAP1 KO guide RNA v2 Complement - AAACCGGGGACTCGGAGACCGACC

YAP1 GCD v2 FWD primer – AGAAAGGGAGGAAGGAAGGA (used for PCR)

YAP1 GCD v1 REV primer – CTGTCGGGAGTGGGATTTG (used for PCR)

YAP1 GCD nested FWD primer – CCCTGAGAGCGAGGACA (used for sequencing)

**Protein Extraction and Western Blot Analysis**

Cells were washed with ice cold PBS and whole cell protein extracts were prepared using RIPA lysis buffer (50mM Tris-HCl pH 8.0, 150mM NaCl, 1mM EDTA pH 8, 1% Triton-X 100, 0.25% sodium deoxycholate, 0.1% SDS) supplemented with Pierce protease inhibitor mini tablets (Thermo Scientific, A32955). Debris was removed from protein extracts by centrifugation at 10,000 x g for 10min at 4 C. Protein concentrations were measured with Pierce Detergent Compatible Bradford Assay reagent (Thermo Scientific). Lysates were separated by electrophoresis on Criterion TGX Precast 10% SDS-PAGE gel (BioRad #5671033 and #5671034) at 100V and transferred to Trans-blot turbo midi format 0.2 μm PVDF (BioRad #1704157) using mixed MW transfer (2.5 A, 25V, 7min) on the TransBlot Turbo Blotting System (BioRad #170-4155). Membranes were blocked with 5% BSA in TBS + 0.2% Tween20 for 2hrs and incubated with primary antibodies diluted 1:1000 in 5% BSA in TBS + 0.2% Tween20 overnight at 4 C. Primary antibodies used included: LKB1 (Santa Cruz, SC-374334), YAP1 (Cell Signaling, 14074S), Actin (Thermo scientific, MA5-11869), a-tubulin (Cell signaling, 2125S), and HDAC1 (Cell signaling, 34589T). Membranes were washed three times in 1X TBS + 0.2% tween20 for 10 min each prior to incubation with secondary antibody. Anti-mouse-HRP or anti-rabbit-HRP secondary antibodies were diluted 1:10,000 in 5% BSA in TBS + 0.2% Tween20 for 1hr at room temperature. After four 10 min washes in TBS + 0.2% Tween20, membranes were subject to Clarity Max Western ECL substrate (Biorad, 1705062S) and imaged with an Amersham Imager 600 RGB (GE Healthcare). Blots were stripped using Restore PLUS Western Blot Stripping Buffer (Thermo Scientific, 46430).

**RNA Isolation and quantitative real time RT-PCR (qRT-PCR)**

Cells were seeded at 0.6 x 10^6 cells/well in a 6 well plate (Corning). Next day cells were replenished with fresh RPMI with 2mM L-Glutamine + 10% FBS or stressed with RPMI without L-glutamine + 10% FBS. After 24hrs of glutamine depletion, RNA was isolated using the Thermo scientific GeneJET RNA Purification kit (Cat# K0731) following the manufacturer’s recommended protocol. mRNA expression for select genes was quantified on a QuantStudio 3 Real-Time PCR System (Applied Biosystems cat# A28136) using the SYBR-based Luna Universal one-step RT-qPCR kit (New England Biolabs, E3005), 105 ng of RNA, and 0.5uM of primer pairs per well. Primers utilized included: human IL6 (forward: AAAGAGGCACTGGCAGAAA; reverse: CAGGCAAGTCTCCTCATTGAA), human CXCL8 (forward: TAGGACAAGAGCCAGGAAGA; reverse: AACTGCACCTTCACACAGAG), human CXCL2 (forward: CAAGAACATCCAAAGTGTGAAGG; reverse: CCATTCTTGAGTGTGGCTATG), human CTGF (forward: ACCTGGAAGAGAACATTAAGAAGG; reverse: TCCACAGAATTTAGCTCGGTATG), human YAP1 (forward: AGCATCTTCGACAGTCTTCTTT; reverse: GTTGTTGTCTGATCGATGTGATTTA), and human PSMB4 (forward: CGAGATGCCCGTTCTTACAA; reverse: CTGTATTTCATTCAAAGCCACTGAT). All primer pairs were designed such that the reverse primer spanned an exon/exon junction. Expression for target genes was normalized to PSMB4.

**RNA sequencing analysis**

Cell lines were seeded at 0.6 x 10^6 cells/well in a 6 well plate (Corning). Next day cells were replenished with fresh RPMI with 2mM L-Glutamine + 10% FBS or stressed with RPMI without L-glutamine + 10% FBS. Total RNA was isolated from all cells 24hrs post glutamine depletion using the Thermo scientific GeneJET RNA Purification kit (Cat# K0731) following the manufacturer’s recommended protocol. Biological replicates of RNA isolated from all cell lines were sent to Novogene for QC analysis, RNA library prep, and sequencing. Subsequent FASTQ files were aligned using STAR (v2.7.10a). Library counts were normalized using the Trimmed Means of M method and batch corrected using COMBATseq prior to performing differential gene expression analysis with DESeq2 (v1.40.2). Pathway enrichment analyses were performed on DEGs using the the GSEA software created by the Broad institute and the R package PathfindR (v2.2.0). Heatmaps were generated using the R package Pheatmap (v1.0.12) and dot plots generated using the R package FlexDotPlot (v0.2.2).

**Code availability**

All custom scripts used for figure generation will be made available upon request.

**Enzyme-linked immunoassays (ELISA)**

Cells were seeded at 0.6 x 10^6 cells/well in a 6 well plate (Corning). Next day cells were replenished with fresh RPMI with 2mM L-Glutamine + 10% FBS or stressed with RPMI without L-glutamine + 10% FBS. Conditioned media was obtained from all cell lines after 24hrs of RPMI + 10% FBS media or 24hrs of RPMI without L-glutamine + 10% FBS media to analyze extracellular levels of IL-6 and CXCL8. The ELISA MAX Standard Set Human IL-6 kit (Biologend Cat# 430501) was used for analysis of IL-6 protein levels in conditioned media following the manufacturer’s recommended protocol. The ELISA MAX Standard Set Human IL-8 kit (Biologend Cat# 431501) was used for analysis of CXCL8 protein levels in conditioned media following the manufacturer’s recommended protocol.

**YAP inhibitor (Verteporfin) experiment**

Cells were seeded at 0.6 x 10^6 cells/well in a 6 well plate (Corning). Next day, cells were replenished with RPMI with 2mM glutamine + 10% FBS or RPMI without L-glutamine + 10% FBS and either DMSO control or 1.5 μM Verteporfin. 24hrs after glutamine depletion, RNA was purified and utilized for qRT-PCR as previously described.

**Whole cell extract glutamine depletion time course experiment**

Cells were seeded at 0.6 x 10^6 cells/well in a 6 well plate (Corning). Next day, RPMI without L-glutamine + 10% FBS was added to the 1hr and 16hr time point cells. 0hr glutamine depleted cells were harvested at start of glutamine depletion for 1hr and 16hr time points. Protein extraction and western blot analysis was performed as previously described.

**Nuclear fractionation experiment**

Cell fractionation performed as previously described (Lee, Y. et al, 2018). Briefly, cells were seeded at 0.6 x 10^6 cells/well (6-well plate, Corning) in RPMI + 10% FBS with or without L-glutamine. At time of harvest cells were washed with 1mL cold PBS and transferred to 1.5mL microcentrifuge tubes, scraping into 1ml cold PBS. After rapid pelleting, cells were resuspended in 400uL cold hypotonic buffer (10mM HEPES pH 7.9, 10mM KCl, 0.1mM EDTA, 0.1mM EGTA, 1mM DTT, and 0.5mM PMSF) and placed on ice for 5 min. Cell membrane disruption was achieved by adding 10% IGEPAL to 0.6% and vortexing followed by rapid pelleting. Supernatant was collected as the cytosolic fraction and remaining nuclear pellet washed twice with cold PBS. Nuclear protein was extracted with 40 μL hypertonic buffer (20mM HEPES pH 7.9, 0.4M NaCl, 1mM EDTA, 1mM EGTA, 1mM DTT, and 1mM PMSF) by rocking tubes for 15min at 4 C and then pelleting samples for 15 min at 13,000 rpm and 4 C. Following SDS-PAGE and Western Blot, ImageJ downloaded from NIH (<http://imageJ.nih.gov/ij>) was used to determine relative cytoplasmic and nuclear YAP1 abundance compared to the loading controls: Tubulin and HDAC1, respectively.

Reference:

Lee, Y., Kim, N.H., Cho, E.S. *et al.* Disheveled has a YAP nuclear export function in a tumor suppressor context-dependent manner. *Nat Commun* **9**, 2301 (2018). https://doi.org/10.1038/s41467-018-04757-w
